# The humoral immune response of the lepidopteran model insect, silkworm *Bombyx mori* L., to microbial pathogens

**DOI:** 10.1016/j.cris.2024.100097

**Published:** 2024-09-18

**Authors:** Abrar Muhammad, Chao Sun, Yongqi Shao

**Affiliations:** a Max Planck Partner Group, Institute of Sericulture and Apiculture, College of Animal Sciences, Zhejiang University, Hangzhou, China; b Analysis Center of Agrobiology and Environmental Sciences, Zhejiang University, Hangzhou, China; c Key Laboratory of Silkworm and Bee Resource Utilization and Innovation of Zhejiang Province, Hangzhou, China; d Key Laboratory for Molecular Animal Nutrition, Ministry of Education, Hangzhou, China

**Keywords:** Silkworm, Humoral immunity, Microbial pathogens, Signaling pathways, Antimicrobial peptides, Phenoloxidase system

## Abstract

Insects are valuable models for studying innate immunity and its role in combating infections. The silkworm *Bombyx mori* L., a well-studied insect model, is susceptible to a range of pathogens, including bacteria, fungi, viruses, and microsporidia. Their susceptibility makes it a suitable model for investigating host-pathogen interactions and immune responses against infections and diseases. This review focuses on the humoral immune response and the production of antimicrobial peptides (AMPs), the phenoloxidase (PO) system, and other soluble factors that constitute the primary defense of silkworms against microbial pathogens. The innate immune system of silkworms relies on pattern recognition receptors (PRRs) to recognize pathogen-associated molecular patterns (PAMPs), which then activate various immune pathways including Imd, Toll, JAK/STAT, and RNA interference (RNAi). Their activation triggers the secretion of AMPs, enzymatic defenses (lysozyme and PO), and the generation of reactive oxygen species (ROS). Collectively, these pathways work together to neutralize and eliminate pathogens, thereby contributing to the defense mechanism of silkworms. Understanding the innate immunity of silkworms can uncover conserved molecular pathways and key immune components shared between insects and vertebrates. Additionally, it can provide valuable insights for improving sericulture practices, developing strategies to control diseases affecting silk production, and providing a theoretical foundation for developing pest control measures.

## Highlights

• Immunity and infection studies revealed a detailed understanding of the silkworm innate immunity.
• The immune system is comprised of conserved components: PRRs, signal transducers, and effectors.
• Different signaling pathways are selectively activated to specific microbial pathogens.
• Humoral immune response is crucial in activating the production of AMPs, melanization, and ROS.
• Understanding the silkworm innate immunity has potential applications in agriculture.

## 1 Introduction

Insects heavily rely on their innate immune system to protect themselves against pathogens because they lack adaptive/acquired immunity (Casanova-Torres Á and Goodrich-Blair, 2013; Lin et al., 2018; Zhang et al., 2015b). This system, which appeared early in insect evolution, consists of physical barriers such as the cuticular integument and peritrophic matrix (PM) (Casanova-Torres Á and Goodrich-Blair, 2013; Davis and Engström, 2012; Erlandson et al., 2019), as well as chemical defenses like hemocytes, antimicrobial peptides (AMPs), and proteins that fight off microbial pathogens (Chen and Lu, 2018; Eleftherianos et al., 2021a, b; Lu and St Leger, 2016). The cuticular integument acts as the first line of defense, creating a barrier against pathogens and other molecules, while the PM protects the gut epithelium and prevents pathogens from entering the hemocoel (Chen and Lu, 2018; Hoffmann, 2003; Lu and St Leger, 2016). If there is a breach in the barrier, the innate immune system coordinates a response to prevent pathogen proliferation within the infected individual (Eleftherianos et al., 2022; Hoffmann, 2003; Lemaitre and Hoffmann, 2007; Nunes et al., 2021; Zhang et al., 2021c). The cellular arm of the immune system involves hemocyte-mediated defense, in which hemocytes undergo morphological and behavioral changes during infection, leading to phagocytosis, nodulation, or encapsulation of pathogens (Eleftherianos et al., 2021a; Jiang et al., 2010; Zhang et al., 2015b). The humoral response, which is the focus of this review, encompasses immune pathways that combat infectious microorganisms by producing AMPs, inducing reactive oxygen species (ROS) synthesis, and generating melanin (Eleftherianos et al., 2022; Park et al., 2018; Zhang et al., 2015b).

Pathogens can infect insects through the gut via ingested food or penetrate the integument through wounds, spiracles, or injections (Chen and Lu, 2018; Eleftherianos et al., 2022; Miyashita et al., 2014). These infections elicit specific immune pathways as part of the humoral immune response, including the Immune deficiency (Imd), Toll, Janus kinase/signal transducer and activator of transcription (JAK/STAT), or RNAi pathways (Chen and Lu, 2018; Lin et al., 2004; Liu et al., 2015; Muhammad et al., 2019a; Wu et al., 2016). A signaling cascade involving pathogen recognition receptors induces intracellular signaling factors that lead to the expression of AMPs and other effector molecules to prevent pathogen proliferation (Aggarwal and Silverman, 2008; Lin et al., 2018; Rämet et al., 2002; Valanne et al., 2011; Waterhouse et al., 2007; Zhang et al., 2021c). The fat body, hemocytes, and gut of insects serve as the primary producers of these molecules (Chen and Lu, 2018; Zhang et al., 2021c). The evolutionarily conserved Imd pathway was first discovered for its role in systemic immunity, activating the production of AMPs in response to Gram-negative bacterial infections (Lee et al., 2017; Lemaitre and Hoffmann, 2007). The Toll pathway was first discovered in *Drosophila* as a type-1 transmembrane receptor functioning in embryonic patterning (Anderson et al., 1985; Hashimoto et al., 1988), but it was later found to play a crucial role in innate immunity, primarily (but not exclusively) in response to Gram-positive bacterial and fungal infections (Lemaitre et al., 1996; Michel et al., 2001; Muhammad et al., 2020). However, it is now evident that these pathways do not exclusively respond to either Gram-negative or Gram-positive bacteria but are also involved in defense against other pathogens including viruses and fungi (Jo et al., 2019; Zambon et al., 2005). The JAK/STAT pathways contribute to gut epithelium renewal, protect the gut from cytotoxic compounds and ROS, and regulate AMP expression (Buchon et al., 2009, 2013; Osman et al., 2012). The RNAi pathway serves as a prominent defense mechanism against viral infections, employing small interfering RNA (siRNA), microRNA (miRNA), and PIWI-interacting RNA (piRNA) cascades (Jiang, 2021; Jiang et al., 2021a). Moreover, the prophenoloxidase (PPO) pathway is responsible for generating cytotoxic compounds upon pathogen detection and facilitates melanin synthesis, which sequesters and eliminates pathogens (Cerenius et al., 2008; Liu et al., 2014).

Lepidoptera, the second-largest order in the class Insecta, is composed of 150,000–180,000 described species (Kawahara et al., 2019). Many Lepidopteran species are serious agricultural pests, while others play crucial ecological roles such as pollination, decomposition, and acting as bio-control agents. Among these, the silkworm, *Bombyx mori* (Bombycidae: Lepidoptera), stands out as one of the most economically important species, having been fully domesticated for silk production in many countries across Asia and Europe (Hu et al., 2023; Li et al., 2017; Lin et al., 2019; Tamilselvan et al., 2017). Beyond its economic importance, the silkworm offers a valuable model organism for various scientific studies, including drug screening, environmental safety monitoring, host-pathogen/microbe interactions, pathology, immunology, and genetic research (Abdelli et al., 2018; Chen et al., 2020; Meng et al., 2017; Muhammad et al., 2022, 2023; Shao et al., 2023). The silkworm's relevance extends to both basic and applied sciences, making it an indispensable resource for researchers. However, the well-being of silkworms is under serious threat from various infectious diseases, including muscardine, pebrine, grasserie, and flacherie, caused by bacterial, fungal, viral, and microsporidian pathogens (Chen and Lu, 2018; Hu et al., 2023; Jiang, 2021; Jiang et al., 2021a; Liu et al., 2015). In China alone, these pathogens cause over a hundred million USD loss in sericulture annually (Li et al., 2017). Therefore, developing novel approaches to control silkworm diseases and enhance their resistance to microbial infections is imperative. Understanding the silkworm's immune response to pathogens is crucial for combating these infectious diseases. This review presents the current advancements in the regulation of the humoral immune system against various pathogens, including bacteria, fungi, viruses, and microsporidia in silkworms. Also, it identifies gaps in the current knowledge and suggests future research directions to advance the field of insect immunology.

## 2 An overview of the silkworm humoral immune system

The humoral immune system plays a crucial role in the innate immunity of silkworms. It enables a quick and effective response to microbial pathogens. In this article, we provide a detailed overview of the essential components and the mechanisms involved in their functions.

### 2.1 Pattern recognition and pathogen detection

**Fig. 1:**
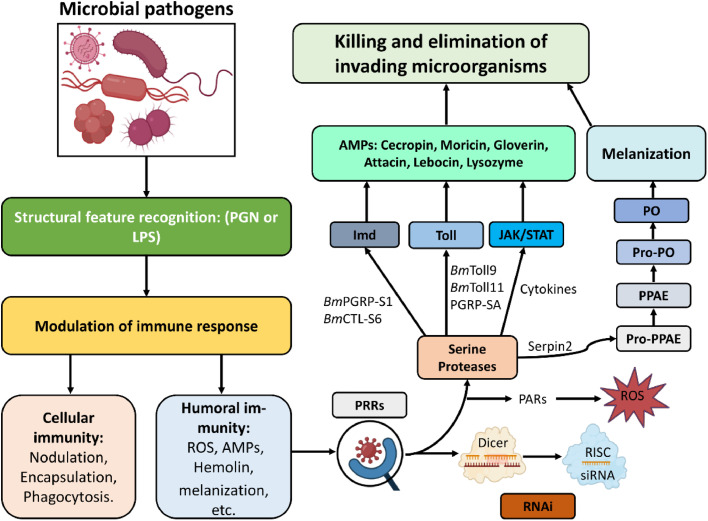
Schematic representation of the modulation of the silkworm immune system upon pathogen challenge. The immune response of silkworms to pathogen challenge involves the recognition of pathogen-associated molecular patterns (PAMPs) by pattern recognition receptors (PRRs), leading to the activation of innate immunity (both cellular and humoral) in silkworms. The humoral innate immune system in silkworms consists of the Imd, Toll, and JAK/STAT pathways, which are responsible for various defense mechanisms against pathogens. These mechanisms include the secretion of antimicrobial peptides (AMPs), production of reactive oxygen species (ROS), activation of phenoloxidase (PO), melanization, and activation of RNA interference (RNAi) pathways targeting different pathogens. The pathway components involved in these processes include serine proteases (SPs), ROS, peptidoglycan (PGN), lipopolysaccharide (LPS), phenoloxidase-activating enzyme (PPAE), and prophenoloxidase (PPO).

The humoral immune response in silkworms begins with the recognition and response to pathogens. This is achieved through pattern recognition receptors (PRRs), which detect pathogen-associated molecular patterns (PAMPs) found on the surface of microbial pathogens (Fig. 1). Silkworm possesses numerous PRRs, each specializing in recognizing different PAMPs (Hou et al., 2011, 2014; Hu et al., 2021; Ito et al., 2021; Ma et al., 2013). Among these, the primary PRRs critical for detecting bacteria, fungi, and viruses and activating the appropriate immune pathways are peptidoglycan recognition proteins (PGRPs), β-Glucan recognition proteins (βGRPs), and C-type lectins (CTLs). PGRPs, which were first discovered in silkworms, are proteins that are highly conserved (Yoshida et al., 1996). They can recognize peptidoglycan (PGN) and activate the Imd and Toll pathways through the nuclear kappa-B (NF-κB) factor (Tanaka and Sagisaka, 2016). In silkworms, researchers have identified multiple PGRPs (12 PGRPs), including short-type (BmPGRP-S1–6) and long-type BmPGRP-L (Tanaka et al., 2008), which play different roles in activating immune responses against different bacterial infections. Some proteins, such as BmPGRP-S3 to S6, have amidase activity. This suggests that they may function as PGRP scavengers (Tanaka et al., 2008). On the other hand, BmPGRP-L6 is responsible for binding to PGN and activating the Imd pathway (Tanaka and Sagisaka, 2016). βGRPs in the immune system of silkworms recognize and respond to bacterial, fungal, and viral infections by binding to β-glucans and trigger the activation of the prophenoloxidase (proPO) system (Tanaka et al., 2008; Wang et al., 2021a; Yoshida et al., 1986). These proteins are developmentally and tissue-specifically regulated, with high expression in the midgut during the larval stage due to constant exposure to microbial pathogens from ingested mulberry leaves (Wang et al., 2021a).

C-type lectins (CTLs) represent another crucial group of PRRs with diverse roles including pathogen recognition, stimulation of proPO activity, cell adhesion, regulation of cellular immunity, and modulation of apoptosis (Rao et al., 2015; Shen et al., 2021). Twenty-three CTLs in silkworms can be grouped into three categories based on their domain architecture and the number of carbohydrate-recognition domains (CRDs): Single-CRDs CTL-S (12 members), Dual-CRDs immulectins (6 members), and CTL-X (5 members) (Rao et al., 2015). These CTLs are highly expressed in the midgut, fat body, and hemocytes and their transcription levels increase significantly upon bacterial (Shen et al., 2021) or viral infection (Mei et al., 2023). They share conserved domains and perform similar functions as CLTs found in other Lepidopteran insects such as *Helicoverpa armigera* and *Manduca sexta* (Wang et al., 2017; Zelensky and Gready, 2005). This indicates a shared evolutionary origin and a role in immunity.

The Toll receptors, which were originally discovered in *D. melanogaster*, contain an extracellular leucine-rich repeat domain and an intracellular Toll/interleukin domain (Cheng et al., 2008; Yu et al., 2020). Silkworms have 14 Toll receptors that are involved in the recognition of PAMPs and the activation of downstream AMPs and proPO pathways (Wu et al., 2010; Yu et al., 2020). BmToll9 and BmToll11 act as ligands of Spätzle (Spz), which in turn activates the Toll pathway (Wu et al., 2010; Yu et al., 2020). The BmToll pathway responds to microbial infection by inducing AMPs such as cecropin A, lebocin, gloverin3, defensin, moricin, and lysozyme (Yang et al., 2018; Yu et al., 2020). Additionally, BmToll also participates in silkworm embryonic development (Cheng et al., 2008).

One Hemolin receptor found in the silkworm genome is responsible for recognizing bacterial lipopolysaccharide (LPS) and lipoteichoic acid (LTA). This recognition leads to the activation of opsonization and melanization reactions during microbial infection (Aathmanathan et al., 2018). Additionally, its expression increases in response to nuclear polyhedrosis virus (NPV) or injection of double-stranded RNA (dsRNA) (Hirai et al., 2004; Tanaka et al., 2008), suggesting that Hemolin is involved not only in bacterial defense but also in antiviral responses and possibly in mechanisms related to RNAi. Furthermore, the silkworm genome is equipped with 3 fibrinogen-related proteins (FREPs), 4 scavenger receptor A (SR-A), 13 SR-B, 1 SR-C, 1 hemocytin, 4 galectins, 1 nimrod A, 1 nimrod B, 2 nimrod C, 1 draper, and 1 Down syndrome cell adhesion molecule (Dscam) each playing distinct roles in immune defense (Ni et al., 2020; Tanaka et al., 2008). These diverse PRRs contribute significantly to the recognition and defense against various pathogens in the silkworm immune system. However, the overlapping functions of multiple PRRs raise the question of functional redundancy, which warrants further investigation to understand their distinct roles and potential interactions within the immune response framework. Also, future studies should employ gene knockdown (RNAi) or knockout (CRISPR/Cas9) approaches to create silkworm strains with targeted deletions of specific PRRs to investigate their unique function.

**2.2 Silkworm signal modulators and transductors**

Once pathogens are detected by PRRs, a sequence of intracellular signaling cascades is triggered to initiate a robust immune response. These signaling pathways involve various signal modulators and transducers, which ultimately culminate in the activation of specific immune responses (Tanaka et al., 2008; Zhang et al., 2021c). Clip domain serine proteases (CLIPs) and serine protease inhibitors (serpins) are essential components of the extracellular cascade that follow pathogen recognition in silkworms. These molecules play critical roles in activating the Toll pathway and regulating proPO activity, which is crucial for melanization and other immune responses (Dong et al., 2019; Lee et al., 2018a). The silkworm genome encodes 15 CLIP genes (BmCLIP1–15) which interact with prophenoloxidase-activating enzymes (BmPPAEs) to regulate the melanization process, a key defense mechanism that encapsulates and neutralizes pathogens (Ma et al., 2013; Tanaka et al., 2008). Silkworms possess 26 serpin genes that inhibit serine proteases (SPs) by forming covalent bonds, thereby regulating the activity of CLIPs and other proteases involved in immune responses (Tanaka et al., 2008; Toufeeq et al., 2019; Wang et al., 2019). Serpins regulate the activity of CLIPs and other serine proteases, ensuring a balanced immune response by modulating Toll and PPO pathways and preventing excessive tissue damage. Dredd, Tak1, FADD, Tab2, IAPs, IKKβ, IKKγ, Ubc13, and Relish are the key signal transducers that propagate the signal and activate AMPs production through Imd pathway against Gram-negative (Tanaka et al., 2008). Similarly, the signal transductors associated with the Toll pathway in silkworms are Spz (3), Toll-like receptors (14), MyD88 (1), Tollip (2), Tube, Pellino, Pelle, TRAF2, ECSIT, Cactus, and Dif/Dorsal (one each). The JNK pathway consists of signal transductors including Hem, JNK, Fos, and Jun (one each), while the JAK/STAT pathway involves PIAS, SOCS, Domeless, and STAT as signal transductors (one each) (Tanaka et al., 2008). The conservation of these signal modulators and transductors in silkworms underscores their vital role in the innate immune response. Understanding the interactions and regulatory mechanisms of these components is crucial for elucidating the complex immune responses in silkworms and has potential applications in enhancing disease resistance in sericulture.

### 2.3 Silkworm effector molecules

After recognizing pathogens through PRRs and modulating signal transducers, immune pathways in silkworms initiate the production of effector molecules. Silkworms have a wide range of effector molecules that contribute to their immune defense against microbial pathogens. One of these molecules is AMPs, which are cationic active peptides that have a strong affinity for interacting with the phospholipid bilayers of bacterial cell membranes (Eleftherianos et al., 2021b; Lemaitre and Hoffmann, 2007; Yang et al., 2018). This interaction destabilizes and permeabilizes the membranes, resulting in cell lysis and death of the pathogen. AMPs in silkworms are low-weight proteins, typically less than 10 kDa, and composed of fewer than 50 amino acid residues (Makwana et al., 2023). These peptides are synthesized in the fat body, the insect equivalent of the liver, and are secreted into the hemolymph, where they exert their antimicrobial effects (Makwana et al., 2023; Nesa et al., 2020; Yang et al., 2011). The silkworm genome encodes 35 AMPs from seven distinct groups, including attacins, cecropins, gloverins, moricins, lebocins, defensins, and enbocins, each exhibiting broad-spectrum antimicrobial activity against various pathogens, including bacteria (both Gram-negative and Gram-positive), fungi, microsporidia, and viruses (Nesa et al., 2020; Tanaka et al., 2008; Yang et al., 2011, 2018).

Furthermore, there are 13 types of cecropins, which are divided into five subtypes. These subtypes include cecropin A (two genes), cecropin B (six genes), cecropin C (one gene), cecropin D (one gene), cecropin E (one gene), and enbocin (two genes) (Cheng et al., 2006; Nesa et al., 2020; Tanaka et al., 2008). Cecropins exert their antimicrobial activity against bacteria and fungi by binding to the pathogen's lipid membrane and inducing pore formation through their hydrophobic C-terminus. The accumulation of cecropin disrupts the membrane integrity and affects cellular electrolyte balance, ultimately leading to cell death (Gregory et al., 2008; Lu et al., 2016; Makwana et al., 2023). Similarly, attacin AMPs (Bmattacin1 and Bmattacin2) inhibit microbial growth by acting on growing cells. They hinder membrane protein synthesis and alter membrane structure and permeability through their hydrophobic C-terminus (Nesa et al., 2020; Sugiyama et al., 1995; Tanaka et al., 2008). Four gloverin AMPs have been identified in the silkworm genome. Among these, Bmgloverin2 has exhibited the ability to inhibit the growth of *Escherichia coli* JM109 and *Pseudomonas putida*. It achieves this by binding to live cells, causing cellular rupture, and subsequently leading to cell death (Tanaka et al., 2008; Wang et al., 2018).

Moreover, the silkworm genome encodes 12 moricin genes, which are effective against a wide range of bacterial and fungal pathogens. Moricins disintegrate microbial membranes through interactions between their C-terminal region and N-terminal amphipathic α-helix (Makwana et al., 2023; Tanaka et al., 2008). Defensins, especially Bmdefensin A and B, with their conserved cysteine residues and cationic nature, illustrate the importance of structural conservation in immune effectiveness (Kaneko et al., 2008; Makwana et al., 2023). The formation of voltage-dependent anion-selective channels highlights a sophisticated method of inducing cell death in pathogens (Makwana et al., 2023). This mechanism not only disrupts the membrane but also interferes with the pathogen's ion homeostasis, underscoring the multifaceted nature of defensin action. Lebocins (Leb1–4) are proline-rich AMPs characterized by the presence of O-glycosylated residues. They exhibit antimicrobial activity against both Gram-negative and Gram-positive bacteria (Furukawa et al., 1997; Yang et al., 2018; Zhang et al., 2021a).

In addition to the diverse array of AMPs, the silkworm genome contains genes for proPO and a proPO inhibitor (BmPO1), which regulate proPO activity in response to pathogen recognition by PRRs such as PGRPs, β−1,3-glucan, βGRP, and serine proteases (Hou et al., 2011; Tanaka et al., 2008; Toufeeq et al., 2019). The proPO system plays a critical role in the immune response by controlling melanization and pathogen elimination. Upon activation, proPO is converted to active phenoloxidase, which catalyzes the production of melanin around invading pathogens, thereby immobilizing and killing them (Hou et al., 2011; Tanaka et al., 2008; Toufeeq et al., 2019). Additionally, silkworms have lysozymes, a group of bacteriolytic enzymes that serve as an early line of defense and actively contribute to their antimicrobial protection (Satyavathi et al., 2018). These enzymes degrade the peptidoglycan layer of bacterial cell walls, causing cell lysis and death. In summary, silkworms produce a diverse array of effector molecules crucial to their immune response. These molecules inhibit microbial growth, disrupt membrane structure and permeability, and regulate processes such as melanization and pathogen elimination, ensuring a robust defense against a wide range of pathogens.

## 3 Silkworm immune response to bacterial infection

**Fig. 2:**
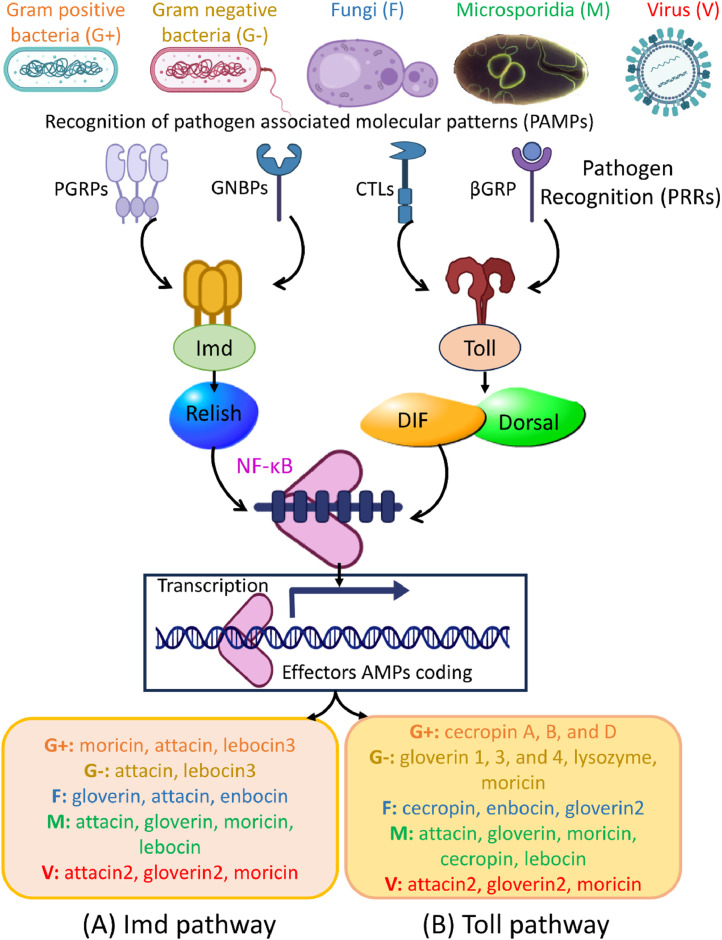
Schematic overview of the (A) Imd pathway and (B) Toll pathway of silkworms in response to microbial pathogens including bacteria, fungi, microsporidia, and viruses. These pathways are initiated upon recognition of microbial components by pathogen recognition receptors (PRRs). This recognition activates the downstream components and the NF-κB transcription factor that eventually trigger the expression of antimicrobial peptides (AMPs) and other effector molecules. See text for further details.

Silkworms have evolved various defense mechanisms to protect themselves against bacterial infections (Table 1). These defenses involve both physical barriers, such as the integument and gut epithelium, as well as the innate immune system (Fig. 1). While bacterial infections typically occur through ingestion of contaminated food or entry via integument wounds, other routes such as respiratory pathways or vector-mediated transmission may also play roles in natural settings (Chen and Lu, 2018; Miyashita et al., 2014). In laboratory settings, the ingestion route is commonly used to simulate infections and investigate immune responses. When bacteria enter through food, they pass through the foregut to reach the midgut (Wang et al., 2022). The midgut PM acts as a barrier to prevent bacteria from entering the hemocoel and modulate the intestinal immune response (Wang et al., 2022; Zha et al., 2021; Zhu et al., 2023). During bacterial infections, the humoral defense mechanisms in silkworms involve the up-regulation of various PRRs such as PGRP-S1, PGRP-S2, PGRP-S3, PGRP-L1, PGRP-L3, βGRP3, and βGRP4, which respond to both Gram-positive and Gram-negative bacteria (Wang et al., 2021b, 2020a; Wu and Yi, 2018) (Figs. 1 and 2). This leads to the expression of AMPs including attacin, cecropin A, cecropin B, gloverin 2, and gloverin 3 as well as ROS, generated from NADPH, to enhance their immune response (Wang et al., 2020a; Zhang et al., 2015a). When BmPGRP-S1 binds to bacterial PGN derived from *Staphylococcus aureus* and *B. subtilis*, the Imd pathway induces the expression of AMPs including moricin, cecropin D, and lebocin3. Silencing of BmPGRP-S1 reduced the expression levels of AMP genes, confirming its role as a PRR in the activation of the Imd pathway during bacterial challenge (Wang et al., 2021b). Similarly, the C-type lectin-S6 (BmCTL-S6) is involved in host immunity against *Micrococcus luteus, E. coli,* and *B. subtilis*. The recombinant CTL-S6 exhibits an affinity for bacterial cell wall components and induces encapsulation, melanization, and stimulation of phenoloxidase (PO) activity. These actions collectively contribute to the immune response against bacterial pathogens (Shen et al., 2021).

**Table 1:** Microbial pathogens infecting silkworms and their host-specific immune response.

Pathogen	PPRs/intracellular signaling factors	Defense response	Reference
E. coli, P. aeruginosa, B. bombysepticus, B. thuringiensis, B. ubtilis, S. aureus	*Scavenger receptor C (SR-C)*	*CecropinA1, Lebocin, Defensin, Gloverin3, and Moricin*	(Zhang et al., 2021b)
*E. coli, M. luteus*	*CTL-S6*	PPO activity	(Shen et al., 2021)
*Staphylococcus aureus*	*Toll9*	NA	(Wu et al., 2010)
*P. aeruginosa* *B. bombysepticus*	*Doux*	*Gloverin 2, Gloverin 3*	(Zhang et al., 2015a)
*E. coli* JM109, *P. putida*	NA	*Gloverin2*	(Wang et al., 2021a)
*E. coli*	*Serine protease SPH-1*	Melanization	(Lee et al., 2018a)
*E. coli, B. bassiana,* and *B. cereus*	*cathepsin l-like*	NA	(Sun et al., 2021)
*E. coli* DH5-Alpha, *S. aureus*	*C-type lectin-domain proteins*	NA	(Rao et al., 2015)
*P. aeruginosa* PAO1	NA	*Gloverin*, PO activity	(Ma et al., 2019)
*B. bombysepticus, Y. pseudotuberculosis*	*PGRPs, C-type lectins, relish, serpins, Toll*, etc.	Generation of ROS, *gloverin2, 3,* and *4, attacin, lysozyme, cecropin A* and *B*	(Wang et al., 2020a)
*E. coli, P. aeruginosa,* *S. aureus, B. subtilis*	*Scavenger receptor class B (SR-B)*	*Attacin1, cecropin A* and *B, defensin, gloverin1, 2, 3,* and *4, lebocin, moricin*	(Zhang et al., 2021a)
*E. coli* DH5α, *S. aureus, B. subtilis*	*PGRP-S1*	*Moricin, cecropin D, lebocin3, attacin*	(Wang et al., 2021b)
*E. coli, M. luteus*	*serpin32*	Regulating PO activity and melanization	(Wang et al., 2019)
*B. thuringiensis*	*PGRP-S6 and S2, PGRP-LB, beta-1,3-glucan4* and *3*	*Gloverin1–3, attacin, lebocin3, lysozyme, moricin*	(Wu and Yi, 2018)
*B. subtilis, S. cerevisiae*	Spz4	Attacin, moricin, gloverin	(Huang et al., 2018)
*Bm*NPV*, E. coli, B. bassiana, M. luteus*	*Rab-related protein genes*	*BmHOP, BmSTAT, BmSOCS2,* and *BmSOCS6*	(Chen et al., 2018)
*Bm*NPV*, N. bombycis, E. coli, S. aureus*	*Relish 1*	*Cecropin B, Attacin,* and *Lebocin*	(Yashwant et al., 2022)
*Bm*NPV	*Serpin2*	Negative regulator of PO activity	(Toufeeq et al., 2019)
*Bm*NPV	*PGRP2–1*	Negative regulator of PTEN	(Jiang et al., 2019)
*Bm*NPV	*Serine protease*	*Attacin, serpin-5, cecropin-D*	(Dong et al., 2019)
*Bm*CPV	PGRP-S2	attacin2, gloverin2, and moricin	(Zhao et al., 2018)
*Bm*NPV	*p38 MAP Kinase* and *Ribosomal S6* *Kinase*	NA	(Muhammad et al., 2019b)
*Bm*NPV	*Toll-6, Toll-7, Toll-8,* and *Toll-9*	*Seroin1, Seroin2*	(Singh et al., 2019)
*Bm*CPV	PGRP-LB, serpin5, serpin28	Attacin1, cecropinA and B, defensin, gloverin1–4, Hemolin, lebocin1–4, moricin	(Kolliopoulou et al., 2015)
*B. bassiana, C. albicans.*	*Serine protease inhibitors of Kazal-type (SPINKs)*	Encapsulation and melanization	(Dong et al., 2021)
*B. bassiana*	Toll signaling pathway	*cecropin A, attacin 1,* and *gloverin 2*	(Geng et al., 2021)
*B. bassiana*	Imd signaling pathway	*enbocin 1, gloverin* *2* and *attacin 1*	(Geng et al., 2021)
*B. bassiana*	JAK/STAT signaling pathway	*storage protein 30K-19G1 (Bmsp 1), attacin 1,* and *cecropin*	(Geng et al., 2021)
*N. bombycis*	*β-glucan recognition protein, β-* *GRP2, PGRP-S3 and PGRP-S4*	*gloverins, lebocins,* and *moricins*	(Ma et al., 2013)
*N. bombycis*	*BGRP1–4, CTL5*	*Lebocin, gloverin, cecropin, attacin*	(Yue et al., 2015)
*N. bombycis*	*serpin 6*	Inhibits PO activity and melanization	(Bao et al., 2019)

Furthermore, BmToll9 and BmToll11, transmembrane proteins belonging to the Toll family, are crucial for the local intestinal immune response in silkworms against infections by *S. aureus* and *E. coli* (Wu et al., 2010). These proteins induce the expression of AMPs (Yu et al., 2020. Interestingly, the Toll pathway in silkworms diverges from its counterparts in other insects by responding to both Gram-positive and Gram-negative bacteria (Wang et al., 2020a; Yu et al., 2020). This dual response capability underscores the adaptability and efficiency of the silkworm immune system. Unlike *Drosophila melanogaster,* where the Toll pathway primarily targets fungal and Gram-positive bacterial infections, in silkworms, this pathway also plays a significant role in gut immunity. This involvement is evidenced by the upregulation of key pathway genes, including Pelle, cactus, and Toll9, following bacterial infection (Wang et al., 2020a). This adaptation suggests a broader functional scope of the Toll pathway in silkworms, enhancing their ability to combat a diverse range of pathogens within the gut environment.

The silkworm immune system employs a variety of scavenger receptors (SRs) to detect and respond to bacterial infections. BmSCRB8, one of these SRs, is constitutively present in immune organs and tissues such as the fat body, gut, and hemocytes. This receptor is crucial for in vivo bacterial clearance, including pathogens such as *E. coli, P. aeruginosa, S. aureus,* and *B. subtilis* (Zhang et al., 2021a). Silencing of BmSCRB8 not only impairs bacterial clearance but also reduces the synthesis of antimicrobial peptides (AMPs) such as attacin, cecropinA, cecropinB, gloverin1, gloverin2, gloverin3, gloverin4, lebocin, and moricin. Consequently, this reduction leads to decreased larval survival upon bacterial infection (Zhang et al., 2021a). Similarly, BmSR-C, another scavenger receptor with a strong affinity towards Gram-positive Lys-type peptidoglycan (PGN), plays a pivotal role in regulating AMP production via the Toll pathway (Zhang et al., 2021b). The presence of BmSR-C highlights the complexity and specificity of the silkworm immune system in recognizing and responding to different types of bacterial infections. The functional diversity of SRs suggests a highly specialized and adaptable immune system capable of mounting effective responses against varied microbial threats.

**Fig. 3:**
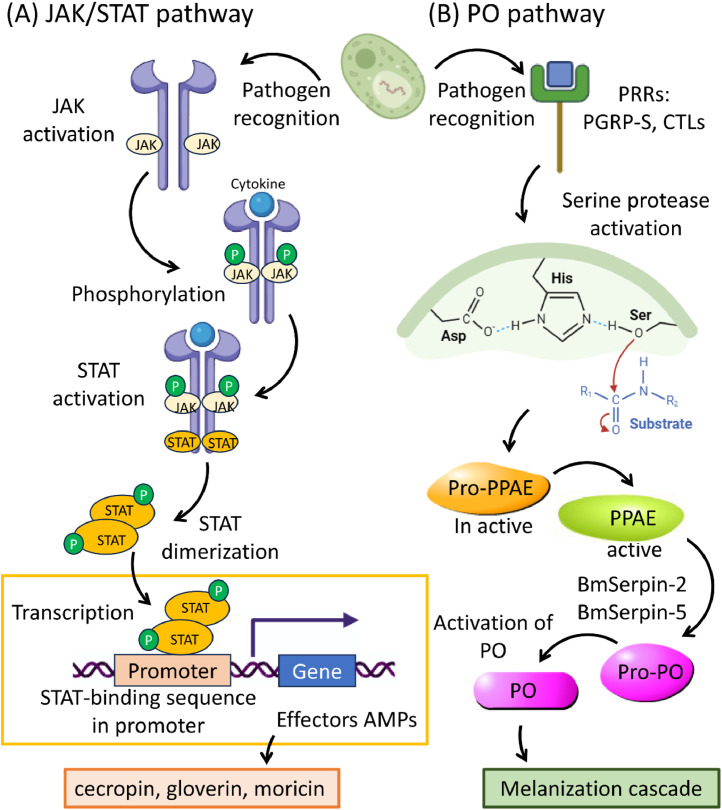
Schematic overview of the (A) JAK/STAT pathway and (B) Phenoloxidase (PO) pathway of silkworms in response to microbial pathogens. Recognition of pathogen-associated molecular pattern (PAMPs) by pathogen recognition receptors (PRRs) triggers the signaling cascades that activate these pathways. JAK/STAT pathway regulates the transcription of antimicrobial peptides (AMPs), while thew PO pathway triggers melanization, and together they play a crucial role in silkworms’ defense against invading microorganisms.

Silkworms challenged with *Bacillus thuringiensis* (Bt) upregulate PGRP-S6, β−1,3-glucan, PGRP-LB, and PGRP-S2, which leads to the induction of gloverin1, gloverin3, gloverin4, attacin, lebocin-3, lysozyme, and moricin (Wu and Yi, 2018). The interaction between these receptors and Bt PAMPs activates the Imd, Toll, and JAK/STAT pathways, resulting in the production of these AMPs (Wu and Yi, 2018) (Fig. 2AB, Fig. 3A). The activation of multiple immune pathways (Imd, Toll, JAK/STAT) in response to Bt infection demonstrates the intricate network of signaling cascades that regulate the silkworms’ immune response. The cross-talk between these pathways ensures a robust and coordinated defense, providing multiple layers of protection. Bmgloverin2 has been shown to inhibit the growth of *E. coli* JM109 and *Pseudomonas putida* by binding to live cells, leading to cell breakage and death (Wang et al., 2018). The mechanism by which gloverins bind to bacterial cells and induce cell rupture suggests a direct interaction with bacterial membranes, a mode of action that is effective and likely to limit the development of resistance. In addition to AMPs like gloverins, silkworms employ serpins as part of their immune defense. Specifically, Bmserpin32 acts as an inhibitor of the extracellular serine protease cascade, exhibiting strong inhibitory activity against trypsin. This serpin is significantly upregulated following infection with *Micrococcus luteus* and *E. coli*, highlighting its role in controlling the proteolytic activity associated with immune responses (Wang et al., 2019). Moreover, Bmserpin2 is implicated in the regulation of melanization, a critical process in the silkworms’ defense against pathogens, demonstrating the multifaceted roles of serpins in immune regulation (Wang et al., 2019) (Fig. 3B).

Furthermore, the midgut immune system of silkworms is a critical component of their defense against bacterial infections. In response to pathogenic bacteria such *as P. aeruginosa, Bacillus bombysepticus, E. coli,* and *Yersinia pseudotuberculosis*, as well as fungi and parasites, the midgut produces reactive oxygen species (ROS) as its primary immune defense mechanism (Wang et al., 2020a). This production of ROS leads to the generation of hydrogen peroxide (H_2_O_2_) and nitric oxide (NO), powerful antimicrobial agents that inhibit the proliferation of pathogens (Chen and Lu, 2018; Hu et al., 2013; Wang et al., 2020a; Zhang et al., 2015a). The importance of ROS in the silkworm immune response is further supported by research on BmDOUX, a key regulator of ROS production. Knockdown of BmDOUX results in a significant increase in microbial proliferation and gut bacterial load, highlighting its essential role in maintaining gut homeostasis by controlling pathogen growth and preventing the overgrowth of resident gut microbiota (Hu et al., 2013; Wang et al., 2020a). This indicates that BmDOUX is not only vital for pathogen restriction but also for the balance of the gut microbiome. This delicate balance is crucial for maintaining gut homeostasis and effective pathogen defense. Overall, the humoral immune response of silkworms to bacterial infection is a complex system that includes PRRs, AMPs, and ROS. This system effectively fights against various pathogens and also helps in maintaining gut homeostasis. Although there have been significant advancements in this area, there is still a need for further investigation into pathogen-specific immune strategies, the interaction between the immune system and gut microbiota, and a deeper understanding of immune regulation mechanisms.

## 4 Silkworm immune response to fungal infection

Silkworms are susceptible to entomopathogenic fungi such as *Beauveria bassiana* and *Aspergillus*, which cause muscardine disease and significant losses in sericulture (Chen et al., 2020; Geng et al., 2021; Hou et al., 2014; Zhang et al., 2022). The infection process begins with fungal spores adhering to the silkworm's cuticle, followed by cuticle penetration and replication within the hemocoel, ultimately leading to the death of the infected individuals (Chen and Lu, 2018; Hou et al., 2014; Lu and St Leger, 2016). The invasion of silkworms by *B. bassiana* occurs rapidly, often within 6–8 h, and the infected individuals display distinctive oily spots, characteristic of white muscardine symptoms. The infected silkworms usually succumb to the infection within 2–3 days (Hou et al., 2011, 2014). The immune response of silkworms to entomopathogenic fungi has been the subject of several studies, aiming to unravel the underlying mechanisms and shed light on this important aspect (Dong et al., 2021; Geng et al., 2021; Hou et al., 2011, 2014; Huang et al., 2018; Lu et al., 2021). Key components of the silkworm's immune response against fungal infection include the activation of signaling pathways such as Toll, Imd, and JAK/STAT and cellular responses like phagocytosis and encapsulation (Dong et al., 2021; Geng et al., 2021).

The activation of key immune-related pathways is crucial for mounting an effective defense against fungal infections in silkworms. These pathways mediate the expression of various AMPs and other immune-related proteins (Hou et al., 2011, 2014; Geng et al., 2021). *B. bassiana* infection activates the Toll signaling pathway and mediates the expression of specific antifungal peptides, including Bmcecropin A, Bmattacin 1, and Bmgloverin 2, while the JAK/STAT signaling pathway mediates Bmstorage protein 30K-19G1 (Bmsp 1), Bmattacin 1, and Bmcecropin A (Geng et al., 2021; Hou et al., 2011, 2014) (Fig. 2). The Imd pathway also plays a crucial role in antifungal defense, and its inhibition has been shown to reduce antifungal activity significantly (Geng et al., 2021; Hou et al., 2014; Lu et al., 2021). Analysis of differentially expressed genes (DEGs) reveals multiple genes involved in the immune response, stress response, and key pathways related to fungal infection (Hou et al., 2011, 2014). Additionally, the temporal characterization of DEGs showed changes in gene expression over time following fungal challenge (Hou et al., 2011, 2014), providing valuable insights into the molecular mechanisms underlying the early immune response of silkworms to fungal infection. However, there is a lack of functional validation for these genes in the early response to *B. bassiana* infection.

The Spz4 gene in silkworms is closely related to Spz4 in *D. melanogaster* and plays a crucial role in the expression of AMPs such as Bmmoricin and Bmgloverin in the integument (Huang et al., 2018). It is important to note that the expression of AMPs regulated by the Spz4 gene may also occur in other tissues, suggesting a broader role in systemic immunity. Additionally, the SPINK7 gene, which is a serine protease inhibitor with three Kazal domains, is involved in regulating the immune defense of silkworms (Dong et al., 2021). It helps in the recognition of fungal infections and promotes hemocyte aggregation, leading to encapsulation, melanization, and inhibition of fungal growth. Moreover, SPINK7 regulates protease activity and inhibits proteinase K from fungal pathogens (Dong et al., 2021), highlighting its multifaceted functions in immune response regulation. BmcecropinA, an important AMP in silkworms, is constitutively expressed in various tissues, including the fat body, silk gland, hemocytes, midgut, and Malpighian tubules. It exhibits strong antifungal activity, as demonstrated by both in vivo and in vitro antimicrobial assays (Lu et al., 2016).

To understand the proteomic changes in silkworms in response to fungal infection, Lu et al. (2021) conducted an iTRAQ-based quantitative proteomic analysis. This approach revealed differentially expressed proteins involved in various biological processes, including the immune response, stress response, and metabolism, in silkworms following infection with *B. bassiana*. One notable finding was the remarkable increase in expression of the BmPGRP-S6 receptor, which corresponded to the activation of various AMPs such as cecropin, gloverin, and attacin. Furthermore, lysozyme proteins, although less known for their antifungal role, showed significant upregulation post-infection, potentially contributing to the defense of silkworms against *B. bassiana*. The analysis also revealed an enrichment of differentially expressed proteins associated with the Toll signaling pathway in the infected individuals. Another interesting finding was the noticeable increase in free fatty acid components, particularly long-chain-fatty-acid–CoA ligases, which are well-known for their antifungal role and might confer resistance against entomopathogenic fungi in infected larvae. Glutathione S-transferases (GSTs), which not only degrade certain insecticides but also some xenobiotics (Muhammad et al., 2021), were another significant pathway upregulated in infected larvae (Lu et al., 2021). However, it is important to note that this study only focused on the early response to *B. bassiana* infection and did not capture the full spectrum of the immune response or the dynamics of protein expression at later stages of infection. Therefore, functional validation of the identified proteins and pathways is necessary to confirm their roles in the silkworm's immune response. Understanding these defense mechanisms is crucial for developing strategies to mitigate the impact of entomopathogenic fungi on the silk industry.

## 5 Silkworm immune response to viral infection

The sericulture industry faces significant annual losses, reaching up to 16%, due to viral infections. The most notable viruses responsible for these infections are BmNPV, BmCPV, BmBDV (bidensovirus), and baculovirus (Ito et al., 2021; Jiang, 2021; Jiang et al., 2021a, 2019) (Table 1). Viral infections typically occur when silkworms ingest contaminated food, allowing the viruses to enter their midgut. This process leads to the destruction of the PM and the subsequent passage into the hemocoel (Lu et al., 2018). Understanding the immune response and defense mechanisms of silkworms against viral infections is crucial for the development of effective strategies to enhance their resistance (Hu et al., 2023; Swevers et al., 2020; Toufeeq et al., 2019). A recent study provides a valuable resource for understanding the genetic basis of antiviral immunity in silkworms (Xia et al., 2024). The silkworm antiviral immune response involves the regulation of various defense mechanisms, including ROS, AMPs, the RNAi pathway, the stimulator of interferon gene (STING) pathway, Toll, Imd, and the JAK/STAT pathway (Ito et al., 2021; Jiang, 2021; Lu et al., 2018; Xia et al., 2024). On the other hand, viruses have evolved to manipulate the immune pathways of their hosts, including the PI3K/Akt and ERK pathways, to evade immune detection and increase viral replication. In silkworms, viruses manipulate host pathways to facilitate infection and enhance their proliferation. These pathways include the PO pathway, the phosphatidylinositol 3-kinase (PI3K)/protein kinase B (Akt) pathway, and the extracellular signal-regulated kinase (ERK) signaling pathway (Hu et al., 2023; Jiang, 2021).

The PRRs, such as BmPGRP-S2 and BmPGRP-S3, respond to BmCPV infection and subsequently trigger immune responses via intracellular signaling cascades and effector proteins, activating both humoral and cellular immune defense mechanisms (Gao et al., 2014; Lu et al., 2018). Overexpression of BmPGRP-S2 improved antiviral activity, decreased BmCPV-mediated mortality, and increased the expression of major immune pathway-related genes (Bmimd and Bmrelish) and AMPs (attacin2, gloverin2, and moricin) (Fig. 2). Moreover, BmPGRP-S2 promoted the expression of the Imd pathway during BmCPV infection, while no significant effect was observed on the Toll pathway (BmSpz and Bmrel) (Jiang et al., 2019; Zhao et al., 2018). Some studies have suggested a potential role of the Toll pathway in silkworms’ antiviral response, however, further investigation is required to elucidate its specific mechanisms (Jiang, 2021). BmCPV infection resulted in the downregulation of BmToll6 and Bmattacin1, while the expression level of β−1,3-glucan was upregulated in infected larvae (Kolliopoulou et al., 2015). The BmSTAT genes of the JAK/STAT pathway in silkworms are crucial for regulating the immune response by controlling the production of downstream effector proteins (Jiang, 2021; Zhang et al., 2016). Although BmSTAT expression was induced by infections with *B. mori* macula-like virus (BmMLV), BmCPV, and BmNPV, the activation of the JAK/STAT pathway varied across different viruses (Zhang et al., 2016). Furthermore, the PO pathway and melanization reaction may also play a significant role in combating viral diseases. For instance, BmNPV infection induced the expression of Bmserpin2 in hemocytes, fat body, and midgut of silkworms, modulating viral infection by activating PO and inhibiting melanization (Toufeeq et al., 2019). In another study, proteomic analysis identified 12 serpins and 6 AMP proteins among the 2368 identified proteins in response to BmNPV infection. Notably, serpin5 and cecropin-D showed a negative regulatory correlation, while other proteins such as attacin, cecropinD-2, cecropin B, and gloverin1 showed upregulation (Dong et al., 2019).

Heat shock proteins (HSPs) have essential functions in cellular stress responses. They stabilize proteins and cellular structures, regulate apoptosis, and modulate signaling pathways. As a result, they play a vital role in protecting cells from stress-induced damage (Jiang et al., 2021b; Kolliopoulou et al., 2015). The Bmhsp19.9 HSP provide protection against extreme temperatures and adverse environmental conditions to silkworms (Wang et al., 2020b). This protein also enhances their resistance to BmNPV infection (Jiang et al., 2021b; Kolliopoulou et al., 2015). The overexpression of Bmhsp19.9 significantly decreased BmNPV DNA content, indicating its role in inhibiting virus proliferation and contributing to antiviral immunity against BmNPV (Jiang et al., 2021b). Additionally, the involvement of the PI3K/Akt pathway in the interaction between silkworms and viral infections has been demonstrated (Jiang, 2021; Jiang et al., 2019). BmPGRP-S2, induced by BmNPV, suppresses the phosphatase and tensin homolog (PTEN), leading to the dephosphorylation of phosphatidylinositol (3,4,5)-trisphosphate (PIP3) and subsequent regulation of the PI3K/Akt pathway (Jiang et al., 2019; Lee et al., 2018b). In the context of viral infections, this activation enhances the antiviral immunity of the silkworm by promoting cell survival pathways that counteract viral-induced apoptosis. As a result, viral replication and spread are limited. Similarly, the transcription factor forkhead box O (FOXO) proteins contribute to optimal antiviral defense by balancing immune activation and preventing excessive inflammation. In silkworms, BmFOXO upregulates BmPEPCK-2 and the expression of autophagy genes such as ATG6/7/8 (Guo et al., 2019; Kang et al., 2021) and inhibits viral replication. Conversely, suppressing BmFOXO decreases the expression of autophagy genes, inhibits apoptosis, and facilitates viral multiplication (Guo et al., 2019; Jiang et al., 2019; Kang et al., 2021). The molecular interactions between Bmhsp19.9 and BmFOXO have important biological implications. These interactions can improve survival rates, reduce viral loads, and enhance resistance to infections in silkworms.

Furthermore, RNAi pathways, including the siRNA, microRNA, and piRNA pathways are major defense strategies against viruses in silkworms and other insects (Jiang, 2021; Kim et al., 2009; Kolliopoulou et al., 2015). Each pathway plays a distinct role in antiviral defense. siRNAs directly target and degrade viral RNA, while miRNAs can either suppress or enhance viral replication depending on their targets. Additionally, piRNAs primarily silence transposable elements while potentially influencing immune responses (Jiang, 2021; Li et al., 2020). Silkworms mount a robust siRNA-mediated defense when infected with viruses like BmCPV and baculovirus, generating a substantial number of siRNAs (Jayachandran et al., 2012; Zografidis et al., 2015). When silkworms are infected with viruses, the viral replication process creates double-stranded RNA (dsRNA) as a byproduct. The enzyme Dicer2 then processes this dsRNA into siRNAs, which are small RNA molecules consisting of 20–25 nucleotides. These siRNAs are subsequently integrated into the RNA-induced silencing complex (RISC), where they join forces with Argonaute 2 (Ago2) (You et al., 2020; Kolliopoulou et al., 2015; Li et al., 2020). Overexpression of BmAgo2, a protein involved in viral RNA cleavage, and BmDicer2, a critical enzyme in recognizing viral RNA, increases the susceptibility of silkworms to dsRNA by facilitating the assembly of siRNA into the RISC complex (You et al., 2020). After BmNPV infection or dsRNA injection (dsBmSPH-1), the upregulation of BmDicer2 in the midgut and hemocytes exhibits moderate inhibitory effects on BmNPV replication, indicating its potential role in silkworm defense against BmNPV invasion (Kolliopoulou et al., 2015; Li et al., 2020). Further studies demonstrate that siRNA-mediated antiviral activity curtails virus replication and mitigates silkworm mortality in BmNPV, BmCPV, and BmBDV infections (Jiang et al., 2017, 2013; Li et al., 2020; Sun et al., 2018). miRNAs have been recognized as vital regulators in the interactions between silkworms and viruses (Jiang, 2021; Jiang et al., 2021a). For example, the host miRNA bmo-miR-2819 suppresses the multiplication of BmNPV by downregulating the viral ie-1 gene (Wu et al., 2019). On the other hand, virus-encoded miRNAs, such as BmNPV-miR-1 and BmNPV-miR-3, enhance virus multiplication by modulating the expression of exportin-5 cofactor Ran and P6.9, respectively, thereby increasing infection rates (Singh et al., 2012, 2014). These miRNAs play important roles in modulating the viral life cycle and the interactions between the host and the virus. Although the role of piRNAs in antiviral defense is less well-defined compared to siRNAs and miRNAs, they may contribute to the immune response by regulating the expression of genes involved in antiviral pathways and require further investigation (Jiang, 2021). In summary, the antiviral defense mechanisms in silkworms involve various pathways, including ROS, AMPs, RNAi, STING, and JAK/STAT pathways. This understanding not only provides insights into the molecular defenses of silkworms but also offers valuable information for developing strategies to combat viral diseases in the sericulture industry. However, further research is needed, especially on less understood elements such as piRNAs, to further strengthen these efforts.

## 6 Silkworm immune response to microsporidian infection

The infection of silkworms by *Nosema bombycis*, the causative agent of pebrine disease, leads to significant losses in the silk industry. Infected larvae display symptoms such as brown melanized spots, stunted development, molting difficulties, dehydration, and ultimately, fatal cytopathic necrosis (Hua et al., 2021; Li et al., 2018; Ma et al., 2013). *N. bombycis* utilizes a specialized injection-like mechanism to evade the host's innate immune defenses during early infection stages. By extruding a polar tube to penetrate the host cell membrane and form sporoplasm, the pathogen establishes secondary infections, compounding the damage (Keeling, 2009; Li et al., 2018). This strategy allows *N. bombycis* to manipulate the host's immune response, energy metabolism, and behavior, exacerbating the disease's impact (Hua et al., 2021; Li et al., 2018). Given the economic implications and the pathogen's natural affinity for silkworms, extensive research has been dedicated to unraveling the immune response mechanisms and the intricate dynamics between *N. bombycis* and its host (Bao et al., 2019; Hua et al., 2021; Ma et al., 2013; Ni et al., 2020).

Upon exposure to *N. bombycis*, gene expression analysis revealed significant changes in various metabolic pathways in silkworms (Ma et al., 2013). These pathways include juvenile hormone synthesis (JH), the melanization pathway (serine protease cascade), as well as cellular (CTL11) and humoral (AMPs) immune. Notably, certain PRRs, such as β-GRP2, PGRP-S3, and PGRP-S4, exhibited significant upregulation, indicating the recognition of *N. bombycis* and the subsequent activation of Toll, Imd, and JAK/STAT signaling pathways (Fig. 2, Fig. 3). Genes associated with the Toll pathway, including Spz1, Spz2, Toll1, Toll9, Toll10, Toll11, Myd88, Tube, and Pelle, were upregulated at different time points. Similarly, genes related to the silkworm JAK/STAT pathway, namely Dome, Hop, and STAT1, also exhibited upregulation upon infection (Ma et al., 2013). These observations indicate the activation of Toll and JAK/STAT pathways in response to *N. bombycis,* resulting in the expression of AMPs such as attacin, gloverin, moricin, cecropin, and lebocin. On the other hand, the Imd pathway did not respond to *N. bombycis* infection. Also, most of the genes associated with the silkworm's phenoloxidase cascade melanization (serine proteases SPs) were downregulated following infection. Initially, certain CLIP serine proteases (CLIP7 and CLIP12) and serpins (SPN1, SPN2, and SPN3) were upregulated, but CLIP15 and eight other serpins were downregulated. Seven out of eight induced CTLs were also downregulated. Key enzymes involved in the melanization process, such as BmPPAE, BmPPAE2, and BmTH, initially showed upregulation followed by downregulation, indicating the suppression of the silkworms’ melanization pathway by *N. bombycis* infection (Ma et al., 2013). This finding aligns with another study that highlights the suppression of host hemolymph melanization by *N. bombycis* infection through the secretion of serpins (NbSPN6) (Bao et al., 2019). The introduction of recombinant NbSPN6 to normal hemolymph inhibited PO activity in a dose-dependent manner while blocking NbSPN6 reversed the inhibitory effect. This confirms the direct targeting of the host's PPAE enzyme by NbSPN6 during pathogen infection (Bao et al., 2019).

The STING-dependent pathway is involved in the innate immune response of silkworms to *N. bombycis* infection (Hua et al., 2021). Transgenic silkworm lines, specifically BmSTING^Δ6bp/WT^ and BmSTING^Δ5bp/WT^, show reduced expression levels of BmSTING and microtubule-associated protein 1 light chain 3 (LC3) and exhibit lower mortality during the early stage of *N. bombycis* infection. Interestingly, BmSTING expression increased with the proliferation of *N. bombycis*. Transgenic silkworms with elevated BmSTING expression showed accelerated mortality, suggesting a potential link between BmSTING and the induction of LC3 as a protective response to microsporidian infection (Hua et al., 2021). Furthermore, there was a notable upregulation and secretion of hemocytin, a hemostasis-related protein, in response to the *N. bombycis* challenge, indicating its role in the immune response against this pathogen (Ni et al., 2020). Further investigation revealed that hemocytin adheres to the surface of the microsporidian, facilitating pathogen agglutination and melanization. Silencing hemocytin expression through RNAi technology significantly increases pathogen proliferation, confirming its pro-inflammatory effects in combating *N. bombycis* infection (Ni et al., 2020).

Furthermore, *N. bombycis* infection profoundly affects the basic metabolism, biosynthesis processes, physiological functions, and developmental processes of the silkworms (Hu et al., 2021; Li et al., 2018; Yue et al., 2015). The infection decreased the expression of genes encoding JH esterase, disrupting JH metabolism and potentially impacting the silkworm's physiological processes and development (Yue et al., 2015). Despite a marked decrease in ATP content, genes involved in ATP synthesis were upregulated, indicating an increased energy demand imposed by the infection. Additionally, elevated levels of lipase-1 promote fat hydrolysis and fatty acid β-oxidation, ensuring an adequate supply of fatty acids and proteins for the pathogen (Li et al., 2018). The elevated expression of protein degradation-related enzymes, such as S-phase kinase-associated protein-1 and the ubiquitin-conjugating enzyme suggests that *N. bombycis* requires host proteins and fatty acids to complete its life cycle (Li et al., 2018). Another study (Hu et al., 2021) has also reported significant changes in gene expression associated with carbohydrate, lipid, nucleotide, and energy metabolism, as well as defense mechanisms after pathogen infection. In summary, *N. bombycis* infection causes various harmful effects on silkworm larvae by influencing the host's energy metabolism, immune response, and behavior. The immune response involves the upregulation of PRRs, activation of signaling pathways such as Toll and JAK/STAT, and the production of AMPs like attacin, cecropin, gloverin, moricin, and lebocin. Understanding the changes in host metabolism during pathogen infection yields valuable insights into vital genes and pathways that are crucial for the survival and growth of the pathogen.

## 7 Conclusion and future perspectives

Silkworms serve as an excellent model for studying insect pathology, immunology, and developmental biology. This review consolidates our current understanding of the innate immunity of silkworms and their responses to pathogen invasion. The humoral immune system plays a crucial role in producing AMPs through the Imd, Toll, and JAK/STAT pathways, which are activated by bacterial, fungal, viral, and microsporidian infections. Key immune mechanisms, including reactive ROS, PO, and melanization, are involved in this response. Additionally, RNAi and the PI3K/AKT pathways play crucial roles in antiviral defense. The adaptability of the silkworm's immune system, which selectively engages different pathways depending on the pathogen, underscores its integrated and flexible nature.

While this review focuses on the humoral immune system, it is worth noting that cellular responses and apoptosis also play a crucial role in shaping the immune response against various pathogens in silkworms. Research on the innate immune system of silkworms presents several potential areas for development. Understanding the molecular mechanisms of silkworm immunity could lead to techniques for modulating immune responses against different pathogens. The application of genetic and biotechnological tools may enable precise editing of the silkworm genome, resulting in the breeding of strains with enhanced immune systems or engineered to effectively express AMPs and other immune-enhancing molecules. AMPs derived from silkworms can be used as therapeutic agents against bacterial infections, paving the way for novel antibiotics or alternative treatments for drug-resistant infections. Additionally, exploring the interplay between the resident microbiome and the immune system could further enhance the silkworm's immune response. Overall, ongoing research on silkworm innate immunity could offer significant theoretical and practical benefits, including enhancing immune responses, increasing silk production, and reducing losses from disease outbreaks.

## CRediT authorship contribution statement

**Abrar Muhammad:** Writing – original draft, Writing – review & editing. **Chao Sun:** Writing – review & editing. **Yongqi Shao:** Supervision, Conceptualization, Funding acquisition, Writing – review & editing.

## Declaration of competing interest

The authors declare that they have no known competing financial interests or personal relationships that could have appeared to influence the work presented in this paper.

## Data Availability

No data was used for the research described in the article.
